# Diagnosis of Upper and Lower Respiratory Tract Bacterial Infections with the Use of Multiplex PCR Assays

**DOI:** 10.3390/diagnostics3020222

**Published:** 2013-03-26

**Authors:** Athanasia Xirogianni, Maria Tsolia, Aliki Voyiatzi, Maria Sioumala, Antonia Makri, Athina Argyropoulou, Olga Paniara, Panayotis Markoulatos, Jenny Kourea-Kremastinou, Georgina Tzanakaki

**Affiliations:** 1 National Meningitis Reference Laboratory, National School of Public Health, 196, Alexandras Avenue, Athens 11521, Greece; E-Mails: axirogianni@esdy.edu.gr (A.X.); msioumala78@yahoo.com (M.S.); kremastinou@esdy.edu.gr (J.K.-K.); 2 School of Health Sciences, Department of Biochemistry and Biotechnology, University of Thessaly, Ploutonos 26 & Aiolou str, Larissa 41221, Greece; E-Mail: markoulatos@bio.uth.gr; 3 Second Department of Pediatrics, University of Athens School of Medicine, P. and A. Kyriakou Children’s Hospital, Thevon & Levadeias str, Athens 11527, Greece; E-Mail: matsolia@ath.forthnet.gr; 4 Penteli’s Children’s Hospital, Microbiology Laboratory, 8 Ippokratous str., Penteli, Attiki 15236, Greece; E-Mails: grace.iliadou@gmail.com (A.V.); tmakri@gmail.com (A.M.); 5 “Evaggelismos” General Hospital of Athens, Microbiology Laboratory, 45–47 Ipsilantou Str, Athens 10676, Greece; E-Mails: gatzea@otenet.gr (A.A.); opaniara@otenet.gr (O.P.)

**Keywords:** ear aspirates, pleural fluids, molecular diagnosis

## Abstract

The investigation of respiratory infections by molecular techniques provides important information about the epidemiology of respiratory disease, especially during the post-vaccination era. The objective of the present study was the detection of bacterial pathogens directly in clinical samples from patients with upper and lower respiratory tract infections using multiplex polymerase chain reaction (PCR) assays developed in our laboratory. Clinical samples taken over a three-year period (2007–2009) and obtained from 349 patients (adults (n = 66); children (n = 283)) with signs and symptoms of certain upper or lower respiratory tract infections, consisted of: bronchoalveolar lavages (BAL, n = 83), pleural fluids (n = 29), and middle-ear aspirates (n = 237). Overall, 212 samples (61%) were confirmed by culture and/or PCR. Among the positive samples, *Streptococcus pneumoniae *(mainly serotype 3) was predominant (104/212; 49.0%), followed by non-typable *Haemophilus influenzae *(*NTHi*) 59/212; 27.8%) and *Streptococcus pyogenes *(47/212; 22%). *Haemophilus influenzae* type b was detected in only three samples. The underlying microbiology of respiratory infections is gradually changing in response to various selective pressures, such as vaccine use and antibiotic consumption. The application of multiplex PCR (mPCR) assays is particularly useful since it successfully identified the microorganisms implicated in acute otitis media or lower respiratory tract infections in nearly 75% of patients with a positive result compared to conventional cultures. Non-culture identification of the implicated pneumococcal serotypes is also an important issue for monitoring pneumococcal infections in the era of conjugate pneumococcal vaccines.

## 1. Introduction

Respiratory diseases represent a major cause of morbidity and mortality among all age groups worldwide. Upper and lower respiratory tract infections involve different bacterial species, which produce indistinguishable signs and symptoms. Infections can be as mild as the common cold, otitis media and pharyngitis, or they can be severe—sometimes even invasive and fatal—as with pneumonia, which is especially the case in the elderly or those suffering from chronic lung disease [1].

Bacteria gain entry into the upper respiratory tract through inhalation, and often establish asymptomatic colonization. From the nasopharynx, the bacteria may spread into the middle ear causing acute otitis media (AOM), or to the respiratory tract and lung parenchyma, causing lower respiratory tract infections.

Nasopharyngeal colonization with *Streptocococcus pneumoniae* and *Moraxella catarrhalis* during the first year of life has been found to occur in 54% and 72% of children, respectively [2], while *Haemophilus influenzae* colonizes 44% of children by the age of two years [3]. At the same time, *S. pneumoniae* is the most important pathogen of AOM, sinusitis, community-acquired pneumonia (CAP), and possibly bacterial bronchitis, whereas *H. influenzae* and *M. catarrhalis* appear to be less common [4]. 

Rapid diagnosis of the causative agent of respiratory tract infections is crucial in reducing morbidity and avoiding excessive and inappropriate antibiotic use which promotes the development of antimicrobial resistance. 

The use of standard culture methods is cumbersome and time-consuming. During the last decade, the introduction of less time-consuming and more sensitive molecular techniques, such as polymerase chain reaction (PCR) assays [5,6], has contributed significantly to diagnosis of infections. Furthermore, the application of multiplex PCR assays (mPCR) for simultaneous identification and serotyping of several respiratory bacterial pathogens seems to be reliable, rapid, and cost effective [7,8].

The aim of the present study was to investigate microorganisms causing respiratory tract infections and evaluate the direct application of four mPCR assays developed in our laboratory, on respiratory tract clinical samples, such as bronchoalveolar lavage (BAL), ear aspirates and pleural fluids. These assays were initially applied on CSF and blood for the diagnosis of meningitis and/or septicemia [7,8,9]. 

## 2. Experimental Section

### 2.1. Patients

Clinical samples collected from 349 patients (children <1–13 years old (n_1_ = 283); adults, 18–70 years old (n_2_ = 66)) with clinical signs and symptoms and/or radiological evidence of upper or lower respiratory tract infection were submitted to the National Meningitis Reference Laboratory from different hospitals throughout Greece during a three-year period (2007–2009). Three groups of patients were involved: *Group A*: patients with otitis media (n = 237), *Group B*: patients with chronic lung diseases who underwent bronchoscopy (n = 83), *Group C*: patients with community-acquired pneumonia (CAP) complicated with parapneumonic effusion (n = 29).

All patients’ records were reviewed. Data recorded included clinical history, including any antimicrobial treatment before presentation, physical findings, laboratory results, and clinical outcome. 

For acute otitis (AOM), the selected study population (*Group A*) presented symptoms and signs of middle-ear inflammation (patients with otorrhea). For those with otitis media with effusion (OME) there was persistent fluid behind the intact tympanic membrane. Clinical examination and myringotomy were performed and were followed by aspiration and culture of middle-ear fluid samples. The criteria for myringotomy in otitis media with effusion included the presence of middle-ear fluid for at least three months.

The patients with possible bacterial lower respiratory tract infection were eligible for the study (*Group B*) after undergoing bronchoscopy and BAL samples were taken by endoscopic procedure. 

The diagnosis of CAP with parapneumonic effusion (*Group C*) was made in adults and children who manifested fever, cough, nasal flaring, tachypnea, retractions, rales, bronchial breathing and decreased breath sounds. Chest radiographs showed indisputable alveolar consolidation with pleural effusion. Pleural fluid was obtained by thoracocentesis or chest-tube placement. 

### 2.2. Clinical Specimens

Clinical specimens from all three groups were collected as soon as possible upon admission. Part of each specimen was processed for routine bacterial culture while the rest was stored in −80 °C for further molecular analysis. 

The 349 samples obtained from both children(n_1_) and adults(n_2_) included: 83 BAL (n_1_ = 30, n_2_ = 53), 29 pleural fluids (n_1_ = 16, n_2_ = 13) and 237 middle-ear aspirates (n_1_ = 237, n_2_ = 0). 

In all three patient groups, only the microorganisms considered true pathogens were included in the analysis, namely, *S. pneumoniae*, *H. influenzae*, Hib and *S. pyogenes*. *S. aureus* was considered a pathogen only in *Group C* patients. 

### 2.3. DNA Isolation

Genomic DNA was obtained from bacterial strains and clinical samples (ear aspirates and pleural fluids) as described previously in detail [7]. 

DNA from BAL was extracted with QIAmp DNA Mini Kit (QIAGEN, Hilden, Germany) according to the manufacturer’s instructions for DNA isolation from tissue with the following slight modification in the first steps of the procedure [10]: 200 μL of the sample were centrifuged at 1,700 ×*g* for 10 min. The supernatant was discarded and 180 μL of buffer ATL (QIAamp DNA mini kit buffer) and 25 μL of proteinase K were added to the pellet. 

### 2.4. Polymerase Chain Reaction (PCR) Amplification

Five multiplex PCR assays were employed for molecular identification of the microorganisms. The first mPCR assay (mPCR-1) was used for the simultaneous detection of *H .influenzae*, *Streptococcus *spp., *P. aeruginosa *and *S*. *aureus* [8]. The second mPCR assay (mPCR-2) was performed for the simultaneous detection of *Neisseria meningitidis*, *S. pneumoniae *and *H. influenzae *type b [7]. For further typing*, *three additional mPCR assays were applied to clinical samples, which were positive for *S. pneumoniae* and *streptococci*: (a) identification of *Streptococcus pyogenes* and *Streptococcus agalactiae*; and, (b) identification of nine serotypes of *S. pneumoniae *(4, 6, 18, 19F, 23F and 1, 3, 4, 19A) using two multiplex PCR assays with a specific primer-pair for each serotype, as described previously (overall specificity 100%) [9]. 

Positive controls from standard strains (5 ng DNA of each species) and negative controls were included in each assay. PCR products were visualized under UV fluorescence following electrophoresis in 2.5% w/v agarose gel stained with ethidium bromide.

#### 2.4.1. mPCR for Simultaneous Identification of Nine Serotypes for *S. pneumoniae*

Briefly, the two multiplex PCR assays were designed as follows: 

##### Multiplex PCR Assay for the Identification of Serotypes 4, 18C, 6, 23F and 19F

The amplification reactions contained: 0.8 mM dNTPs, 1× DyNAzyme II buffer and 1.5 U Taq polymerase (DyNAzyme II Hot start, Finnzymes, Finland), 3 mM MgCl (ABgene, Surrey, UK), and primers for serotypes 4, 18C, 6, 23F and 19F in concentrations of 0.6 μΜ, 1.2 μΜ, 0.2 μΜ, 0.4 μΜ and 0.8 μΜ, respectively [11] and 6 μL DNA template in a total volume of 25 μL. PCR conditions were: 95 °C for 10 min; 5 cycles of 95 °C for 50 s, 63 °C for 35 s, 68 °C for 1.15 min; 5 cycles of 95 °C for 50 s, 61 °C for 35 s, 68 °C for 1.15 min; 23 cycles of 95 °C for 50 s, 59 °C for 35 s, 68 °C for 1.30 min (RoboCycler, Stratagene).

##### mPCR Assay for the Identification of Serotypes 19A, 14, 3 and 1

The amplification reactions contained: 0.8 mM dNTPs, 1.2× DyNAzyme II buffer and 1.5 U Taq polymerase (DyNAzyme II Hot start, Finnzymes, Finland), 3.5 mM MgCl (ABgene, Surrey, UK), and primers for serotypes 19A, 3 and 1 in concentrations of 0.8 μΜ, 0.4 μΜ, and 0.4 μΜ, respectively [11] and 6 μL DNA template in a total volume of 25 μL. For serotype 14, a new primer pair was designed (sp-T14F 7749: 5'-AGAAGTTTGTTAGACTGGGACGGA-3' and sp-T14R 7749: 5'-CGTGTCGCATTGCTACCCGATCTA-3') in *wzy *gene with FastPCR software [12]. A concentration of 1 μΜ was used in the mPCR reaction.

PCR conditions were: 95 °C for 10 min; 5 cycles of 95 °C for 50 s, 63 °C for 40 s, 68 °C for 1 min; 5 cycles of 95 °C for 50 s, 61 °C for 40 s, 68 °C for 1 min; 23 cycles of 95 °C for 50 s, 59 °C for 40 s, 68 °C for 1.30 min.

The overall specificity of the assay was 100% [9]. 

#### 2.4.2. mPCR for Simultaneous Identification of *S. pyogenes* and *S. agalactiae*

For the detection of *S. pyogenes*, oligonucleotide primers were newly designed on the *spy1258* gene [13] using Primer3 software [14] For the detection of *S. agalactiae*, oligonucleotide primers were used as previously described [15] with a slight modification by deletion of some nucleotides of the primer’s 3' end in order to avoid primer dimers (Table 1). 

**Table 1:** Oligonucleotide primers used in multiplex PCR assay for the identification of *Streptococcus pyogenes *and *Streptococcus agalactiae*.

***Microorganism ***	***Pri Sequence (5'–3')***	***Gene***	***Product *** ***size (bp)***	***Publication***
***S.pyogenes***	**SpyF:** ACT CTG GAT GAT TTG TAC CG **SpyR:** TCA GTG GTT TCT TGA TAG CC	***Spy1258***	314	This study
***S.agalactiae***	**CFBS:** ATG ATG TAT CTA TCT GGA ACT CT **CFBA:** CGC AAT GAA GTC TTT AAT TTT TC	***cfb***	259	modified by [15]

The amplification reactions contained: 0.1 μΜ of each of SpyF/R primer (VBC, Hamburg, Germany); 0.8 μΜ of each of CFBA/CFBS primers; 0.8 mM dNTPs (ABgene, Epsom, UK); 1 U Hot Start Taq (Finnzymes Finland); 1.4× reaction buffer; and 5 μL of DNA template in a total volume 25 μL. PCR conditions were: 95 °C for 10 min; 10 cycles of 95 °C for 20 s, 58 °C for 20 s, 68 °C for 40 s; 25 cycles of 95 °C for 20 s, 56 °C for 20 s, 68 °C for 40 s; 68 °C for 5 min in an Apollo thermal cycler (Apollo ATC 201, CLP Inc. USA).

The sensitivity and specificity were 98% and were tested with DNA extracted from 332 collected bacterial strains including: *S. pyogenes* (n = 115), *S. agalactiae* (n = 40), *S. pneumoniae* (n = 147) and *Streptococcus* spp. (n = 30). In order to evaluate the sensitivity of the assay, serial dilutions of spectrophotometrically quantified DNA (5 ng–5 pg/per reaction) from each species were tested (data not shown). 

## 3. Results and Discussion

### 3.1. Sample Type and Bacterial Detection

In total, 212 of 349 (61%) clinical samples were positive either by culture and/or by PCR for the tested microorganisms. Of those, 51/212 (24%) were culture positive, while 160 clinical samples (160/212; 75.1%) were culture negative–PCR positive for the microorganisms S*. pneumoniae*, *S. pyogenes* and *H. influenzae *(Table 2). 

**Table 2:** Detection of bacterial DNA in clinical samples according to the organism involved, patient category and age group.

CHILDREN (n = 181)							
	*S. pneumoniae* (n = 104)		*S. pyogenes* (n = 47)		*H. influenzae * (n = 62) Hib (n = 3), NTHi (n = 59)		
	PCR (+) Culture (+)	PCR (+) Culture (−) *	PCR (+) Culture (+)	PCR (+) Culture (−) *	PCR (+) Culture (+)	PCR (+) Culture (−) *	PCR (−) Culture (+)
**Group A** (n = 135)	1	59	9	30	24	12	0
**Group B** (n = 31)	1	10	0	0	10	10	0
**Group C** (n = 15)	1	11	1	2	0	0	0
**ADULTS (n = 32)**							
**Group B** (n = 21)	1	15	0	0	1	2	2 #
**Group C** (n = 11)	0	5	2	3	0	1	0
**TOTAL**	**4**	**100**	**12**	**35**	**35**	**25**	**2**

Group A: AOM, Group B: Patients with chronic lung disease, Group C: patients with community-acquired pneumonia and parapneumonic effusion. * Only these 2 specimens were PCR (−) culture (+).

Overall, *S. pneumoniae *was identified in almost half of the samples; 104/212 (49%), as a sole pathogen or co-existed with other bacteria in both adults 21/32 (65.6%) and children 83/180 (46.1%). The second most common isolate was *H. influenzae *(NTHi) (59/212; 27.8%) while, *S. pyogenes *was detected in lower proportions 22% (47/212). *H. influenzae* type b (Hib) was found in 3 samples (1.4%) all obtained from children (2 in *Group A* and 1 in *Group B*). 

According to group samples, *S. pneumoniae* was the predominant microorganism detected in ear aspirate samples (*Group A*) (60/213; 28.1%) followed by *S. pyogenes *(39/213; 18.3%) and *H. influenzae* (36/213; 16.9%). 

In *Group B*, the data obtained varied significantly between adults and children: *H. influenzae* was the prevalent microorganism among children’s positive BAL samples (20/31; 64.5%), followed by *S. pneumoniae* (11/31; 35.5%). In contrast, *S. pneumoniae *(16/21; 76.2%) predominated in adults’ BAL samples and *H. influenzae *(*NTHi*) was also detected in a lower proportion (5/21; 23.8%).

For *Group C *(patients with CAP complicated with parapneumonic effusion), the predominant microorganism was *S. pneumoniae* in both children (12/15; 80%) and adults (5/11; 45.5%). *S. pyogenes* (5/11; 45.5%) followed by *H. influenzae *(1/11; 9.1%) were detected in pleural fluids from adults, while *S.pyogenes* was detected in 3/15 (20%) in children (Table 2). There were no cases of staphylococcal parapneumonic effusion identified.

### 3.2. Pneumococcal Serotype Distribution

Serotype distribution of all clinical samples positive for *S. pneumoniae *are summarized in Table 3. 

**Table 3:** Pneumococcal serotypes according to patient category and age group.

Children				Adults		Total
Serotype	Group A	Group B	Group C	Group B	Group C	
**1**			1			**1**
**3**	11		6		3	**20**
**6**	3	5				**8**
**14**	1	1				**2**
**18**	2			3		**5**
**19A**	4		1		2	**7**
**19F**	10	1				**11**
**23F**	1					**1**
**6/18**		1		1		**2**
***subtotal***	***32***	***8***	***8***	***4***	***5***	***57***
Other serotypes	28	3	4	12	0	**47**
**TOTAL**	**60**	**11**	**12**	**16**	**5**	**104**

In children, among the positive specimens which were further typed, serotype 3 was predominant (11/32; 34.4%) in *Group A* (children with AOM) followed by 19 F (10/32; 31.2%) and 19A (3/32; 12.5%). In contrast, among the *Group B* specimens, serotype 6 predominated (5/8; 62.5%), while in *Group C* the majority of the serotypable specimens also belonged to serotype 3 (6/8; 75%). 

Among the adults’ clinical specimens, serotype 18 was predominant in *Group B* specimens while, serotype 3 predominated in *Group C* clinical specimens positive for *S. pneumoniae.*

Interestingly, serotype 4 was not detected in either children or adults. 

## 4. Discussion

During the past decade, positive PCR results from ear aspiration specimens have been taken as evidence for the presence of bacterial DNA in middle-ear effusions. PCR assays have been shown to be more reliable, rapid, and more sensitive than cultures [16,17,18]. In the present study, the mPCR assays detected at least one microorganism in a large proportion of ear aspirate samples (*Group A* patients). 

*S. pneumoniae *remains an important cause of acute otitis media worldwide [19,20], even in the post-PCV7 era, due to the emergence and spread of other serotypes not included in the aforementioned vaccines, such as serotype 19A and, possibly, serotype 3 [21]. Our results come to an agreement with these observations since *S. pneumoniae* was detected in the majority of positive samples while the majority of them were serotype 3, a serotype which is not included in the PCV-7. This is likely due to serotype replacement, since PCV7 vaccine was introduced in Greece in 2004 and was officially included in the national immunization program in 2006.

*H. influenzae *is considered to be the second most frequent pathogen of AOM [20] or OME [17,22] and it was identified in a considerable proportion of our patients. The low percentage of samples positive for *H .influenzae* type b is not unexpected, since the introduction of the conjugate vaccine seems to decrease nasopharyngeal colonization and consequently respiratory and invasive disease associated with this pathogen [1]. 

Finally, *S. pyogenes* continues to be an important pathogen in AOM among older children with higher local aggressiveness manifested by lower rates of fever and higher rates of tympanic perforation and mastoiditis [23]. 

In *Group B* patients, *S. pneumoniae *was detected in a similar rate in both children and adults. This is comparable to previous studies in which it was found in 28% of BAL samples [24]. *H. influenzae, *the second most prevalent microorganism in this group, was detected with a higher rate in children than adults. According to the clinical symptoms, the high percentage in children indicates infection, whereas the lower range of detection in adults might reflect the lower risk of colonization in adult patients [1].

BAL examination may be particularly beneficial for the diagnosis of lower respiratory tract infection among patients who are not responding to the initial antibiotic treatment, in spite of the minimal risk of contamination by the oropharyngeal flora [25,26]. Although fiber-optic bronchoscopy to obtain BAL samples is designed to avoid the colonizing flora of the upper airways, the samples can potentially be contaminated with the oropharyngeal flora by the bronchoscope itself and yield false positive microbiological results [24]. For this reason, all samples positive for *Streptococcus *spp.—streptococci other than *S. pneumoniae* or *S. pyogenes—*were evaluated as normal flora and not as pathogens and were thus excluded from the present study. However, the clinical significance of identification of true pathogens such as *S. pneumoniae *or *H. influenzae *in BAL specimens by PCR in patients with negative cultures remains to be elucidated. 

In *Group C *(patients with pneumonia complicated by parapneumonic effusion), the mPCR assays successfully detected the causative microorganism in 22 out of 26 culture-negative pleural fluid samples. As already indicated in previous studies [27,28], our results revealed *S. pneumoniae *as the most common pathogen. The prevalent serotypes found in this group were serotype 3, 19A and 1, all of which are non-PCV7 serotypes yet are included in the PCV13 vaccine. Although PCV7 clearly reduced the incidence of invasive pneumococcal disease (IPD), there was an increase in pneumococcal pneumonia with empyema in some countries even before the introduction of immunization [29,30].

Because *S. pneumoniae *was found as the predominant species in all three patient groups, serotype identification in the absence of the isolate is of great importance, since it was found that, in the post-PCV7, era non-PCV7 types seem to be predominant.

For this reason, application of PCR assays is crucial, successfully identifying microorganisms causing upper and lower respiratory tract infection in a high proportion of culture-negative clinical samples, especially those from a normally sterile site, such as pleural fluid or ear aspirates, and providing a definite diagnosis. Previous antibiotic treatment may, in part, account for the observed low yield of the bacterial cultures.

## 5. Conclusions

With the use of mPCR assays in this study the causative pathogen was identified in a much higher proportion of patients with different respiratory infections compared to conventional cultures. Accurate diagnosis and pathogen identification is important for patient management and targeted antibiotic treatment. Improved diagnosis is also important for following the effects of new conjugate vaccines on the epidemiology of pneumococcal respiratory infections. 
